# Policy for reducing unplanned pregnancies and repeat unplanned pregnancies rates in Israeli Defense Force

**DOI:** 10.1186/s13584-019-0292-x

**Published:** 2019-02-04

**Authors:** Adi Kuperman-Shani, Tarif Bader, Elon Glassberg, Vered Klaitman, Israel Defense Forces (IDF), Medical Corps

**Affiliations:** 1 Medical Corps, Israel Defense Forces, Hashita 130, Omer, Israel; 2 Surgeon General’s Headquarters, 1101 Wootton Pkwy, Rockville, 20852 MD USA; 3 Department of Military Medicine, Hebrew University, Jerusalem, Israel; 4 Bar-Ilan University Faculty of Medicine, Safed, Israel; 5 The Uniformed Services University of the Health Sciences, Bethesda, MD USA

**Keywords:** Termination of pregnancy, Unintended pregnancy, Military service, Reproductive health, Family planning

## Abstract

Israel has compulsory military service, beginning at the age of 18. Women serve about two years and men for about three years. However, de facto only some of the potential service entrants are recruited. Among women, those who enlist are mainly secular Jews who are unmarried; among men, most of the ultra-Orthodox Jews do not enlist. In addition, only a fraction of the recruits chooses to turn the military service into a career and sign up for additional service as professional military personal (officers and non-commissioned officers). Thus, military personnel are not representative of the general Israeli population, even after controlling for age.

The rate of pregnancies among female soldiers (obligatory service) in the Israeli army is low, but almost all pregnancies in this group are unplanned and most result in termination of pregnancy. An unplanned pregnancy carries a direct impact on the service of that female soldier and consequently on the military’s routine.

In a recent article in the Israel Journal of Health Policy Research (IJHPR), Rottenstreich et al. (IJHPR 7:42, 2018) describe a retrospective cohort study designed to evaluate the prevalence and risk factors for repeated unintended pregnancies among this population of female soldiers.

This commentary presents the current IDF policy intended to further reduce unplanned pregnancies and repeat unplanned pregnancies rates. We also suggest additional tools to support evidence-based strategy planning in this field.

The rate of unintended pregnancy (UP) is a widely used measure of a populations’ reproductive health. Numerous studies have demonstrated an association between unintended pregnancies and a range of negative health, economic, social, and psychological outcomes on the individual women, their children and the society as a whole [1–3].

Reducing the rate of unplanned pregnancies is well established public health objective.

In a recent IJHPR article, Rottenstreich and colleagues report on the prevalence of Recurrent Unintended Pregnancies (RUP) among young unmarried women during their obligatory service in the Israeli military. The authors describe RUP rates of 22% (389/1720) during their study period of 2 years (2013–2015), calculated out of all women with unintended pregnancies who had a follow up period of at least a year, and specify those rates as being high. Risk factors associated with those pregnancies are also defined [4].

That IJHPR article is complementary to a prior article by Rottenstreich and colleagues that described the prevalence of and variables associated with UP among the same study population [5].

We are thankful for the authors for their role in raising awareness of this important issue.

Over the years, a worldwide decline in the rate of UP has been observed [6]. Such a trend was also observed in Israel - both in the general population [7] and specifically in the Israel Defense Forces (IDF). In fact, UP rates in the IDF declined from 2.15% in 2003 to 1.33% in 2015. Indeed, these changes were described by Rottenstreich and colleagues in their aforementioned article [5].

Nevertheless, unintended pregnancies continue to pose a global and a national public health burden and have a major impact on the service of women in the IDF.

Unfortunately, estimating the true incidence of UP at the national or regional level is challenging.

Common definitions are not available and there is great variability across countries in the way UP is measured. In many cases, the prevalence is estimated, based on retrospective surveys (subject to major biases) or abortion rates (which are themselves inaccurate) [8].

Despite these definitional and measurement challenges, Sedgh et al. have examined sub-regional, regional, and global rates and trends of reported abortion incidence between 1990 and 2014, and abortion rates in subgroups of women. A great deal of variation was found between different regions of the world [9].

Bearak et al., 2018, developed a Bayesian hierarchical time series model, whereby the unintended pregnancy rate is a function of the distribution of women across subgroups (defined by marital status and contraceptive needs and use), and of the risk of unintended pregnancy in each subgroup. This model allowed for more accurate estimation, but still showed vast variation between regions and countries [10].

Attempting to compare Repeat Unintended Pregnancies (RUP) rates and prevalence is even more problematic - being subject to recall biases, social and cultural sensitivities and more.

A Finish nationwide retrospective register study examined trends in teenage termination of pregnancy (TOP) rates between 1987 and 2009. This study found that the incidence of repeat TOP increased significantly between 1993 to 2009 and that the proportion of RUPs among 18- to 19-year-olds teens reached 19.2% at 2009 [11].

A retrospective study using data from the Scottish (Grampian) TOP Database’ (1997–2013) showed that 23.4% of women who had an initial TOP went on to go through a repeat termination. Women who had gone repeat terminations were more likely to be aged under 20 years at the time of the initial termination [12].

It is especially difficult to assess the true amount of RUP in Israel, as the information available is derived from indirect measures of the TOP rate and is affected by numerous factors, including social and religious sensitivity. Data collected by the State of Israel Central Bureau of Statistics showed that in 2014, 20% of women aged 24 and under had a history of at least one termination of pregnancy [7].

It is also important to note that Israel has compulsory military service, beginning at the age of 18. Women serve about two years and men for about three years. However, de facto only some of the potential service entrants are recruited. Among women, those who enlist are mainly secular Jews who are unmarried; among men, most of the ultra-Orthodox Jews do not enlist. In addition, only a fraction of the recruits chooses to turn the military service into a career and sign up for additional service as professional military personal (officers and non-commissioned officers). Thus, military personnel are not representative of the general Israeli population, even after controlling for age.

In face of the above, it is unclear why the authors chose to declare the RUP rates in the IDF as “high”. Furthermore, the authors chose to calculate the percentage of RUP out of those cases for which there was at least a one-year follow-up, rather than out of all unwanted pregnancies. Such a calculation would have brought the RUP rate even lower- to 16% (389/2365). So, the actual rate of RUP is lower than that presented.

A distinction between women who had a history of an unintended pregnancy or RUP before joining the IDF and those for whom these events took place while they were on active duty (not included in the analysis) would have also been of interest.

Calculating UP and RUP rates is even more complicated since minor portion of IDF service women may terminate their pregnancies privately and without the knowledge of the IDF medical corps. It is our belief that this phenomenon is more common amongst soldiers from higher socioeconomic classes. The proportion of RUP’s among this group is obviously unknown.

Regardless of the exact RUP rates and with the goal of further reducing unintended pregnancy rates, the IDF medical corps has taken several groundbreaking steps over the recent years. We believe (though unfortunately it is difficult to quantify the effect) that these measures have had a substantial influence on unintended pregnancies prevalence and contributed to its decline. The IDF (a joint effort by the personnel branch and the medical corps) has established a “coping and support center” a confidential center, consisting of an all-female staff of social workers/psychologists and gynecologists who provide support, guidance, counseling and medical care for soldiers coping with an unintended pregnancy.

Some of the pregnant soldiers choose to keep the pregnancy (resulting in discharge from the service) and some choose to terminate the pregnancy. For those who choose to terminate the pregnancy, additional psychological support is offered, along with assistance in returning to duty and to their units, while maintaining their privacy.

Education is a key step in reducing the number of unwanted and unplanned pregnancies. Several risk factors for teenage unintended pregnancies across the world, in Israel and in the IDF have been identified. At risk populations include first generation immigrants, women who have not graduated from high school and those who come from lower socioeconomic backgrounds [13–15]. Thus, educational programs focusing on high-risk populations are held during relevant basic trainings.

Educational efforts should begin during adolescence and the high school years (even before sexual activity begins). Currently there is no obligatory national sexual education plan in place in Israeli high schools as part of an orderly educational program.

Improving the availability of contraceptives is an important component, as well. Adjustments have been made in the IDF over the last few years to support this goal. These include the introduction of the long acting reversible contraceptives (LARC) by the “coping and support center” as primary and secondary prevention [16].

Uppermost importance is devoted to secondary prevention measures. LARC is presented as a first line option and provided by the center to female soldiers presenting with unintended pregnancy choosing to terminate the pregnancy or to those who had an unintended pregnancy in the past, regardless of other risk factors.

## Conclusions

Despite the reduction and the current low rates of UP and RUP in the IDF, this remains a public health concern and the focus of on-going multidisciplinary efforts.

The complexity and difficulty of comparing data between countries and societies enhances the importance of building a comprehensive national database to allow for periodic estimation of the incidence of UP (that will not have to be based on the rates of pregnancies terminations) and to encourage research on sexual behavior, contraceptive use and contraceptive failure studies.

Such databases will allow policy makers (both in the national health system and in the IDF) to monitor the prevalence of unwanted pregnancies, establish interventions and evaluate their impact on UP rates and their outcomes.

A national sexual education program focusing on high school students is key to further reducing UP rates and to building a healthier society.

## Abbreviations

IDF: Israel Defense Forces
LARC: Long acting reversible contraceptives
RUP: Repeat Unintended Pregnancies
TOP: Termination of pregnancy
UP: Unintended Pregnancies

## References

[1] Orr ST, Miller CA, James SA, Babones S (2000). Unintended pregnancy and preterm birth. Paediatr Perinat Epidemiol.

[2] Taylor JS, Cabral HJ (2002). Are women with an unintended pregnancy less likely to breastfeed?. J Fam Pract.

[3] Kost K, Lindberg L (2015). Pregnancy intentions, maternal behaviors, and infant health: investigating relationships with new measures and propensity score analysis. Demography.

[4] Rottenstreich M, Sela HY, Loitner L, Smorgick N, Vaknin Z (2018). Israel journal of health policy research recurrent unintended pregnancies among young unmarried women serving in the Israeli military. IJHPR.

[5] Rottenstreich M, Loitner L, Dar S, Kedem R, Smorgick N, Vaknin Z (2017). Unintended pregnancies among women serving in the Israeli military. Contraception [Internet].

[6] Finer LB, Zolna MR (2016). Declines in unintended pregnancy in the United States, 2008– 2011. N Engl J Med.

[7] Legaly induced Termination of pregnancies 1990–2016. Israely Ministry of Health, department of information. Accessed 24 July 2018. 2018; Available from: https://www.health.gov.il

[8] ESHRE Capri Workshop Group*. Why after 50 years of effective contraception do we still have unintended pregnancy? A European perspective ESHRE Capri Workshop Group. Hum Reprod [Internet]. 2018 [cited 2018 Aug 1];33(5):777–783. Available from: https://academic.oup.com/humrep/article-abstract/33/5/777/4967895 10.1093/humrep/dey089 29659848

[9] Sedgh G, Bearak J, Singh S, Bankole A, Popinchalk A, Ganatra B (2016). Abortion incidence between 1990 and 2014: global, regional, and subregional levels and trends. Lancet.

[10] Bearak J, Sedgh ScDG, Bearak J, Popinchalk A, Alkema L, Sedgh G. Global, regional, and subregional trends in unintended pregnancy and its outcomes from 1990 to 2014: estimates from a Bayesian hierarchical model. Artic Lancet Glob Heal [Internet]. 2018 [cited 2018 Aug 3];6:380–389. Available from: www.thelancet.com/lancetgh 10.1016/S2214-109X(18)30029-9 PMC6055480 29519649

[11] Leppälahti S, Gissler M, Mentula M, Heikinheimo O (2012). Trends in teenage termination of pregnancy and its risk factors: a population-based study in Finland, 1987-2009. Hum Reprod.

[12] Mccall SJ, Flett G, Okpo E, Bhattacharya S. Who has a repeat abortion? Identifying women at risk of repeated terminations of pregnancy: analysis of routinely collected health care data. 2015 [cited 2018 Sep 29]; Available from: http://srh.bmj.com 10.1136/jfprhc-2014-101059 26644146

[13] Sedgh G, Finer LB, Bankole A, Eilers MA, Singh S (2015). Adolescent pregnancy, birth, and abortion rates across countries: levels and recent trends. J Adolesc Heal [Internet].

[14] Sikron F, Wilf-Miron R, Israeli A (2003). Adolescent pregnancy in Israel: a methodology for rate estimation and analysis of characteristics and trends. Harefuah.

[15] Yagil Y, Elran E, Tarchitzky O, Levy Y, Ashkenazi I (2005). Unplanned pregnancies among women soldiers in the IDF--an overview. Harefuah.

[16] Secura GM, Madden T, McNicholas C, Mullersman J, Buckel CM, Zhao Q (2014). Provision of no-cost, long-acting contraception and teenage pregnancy. N Engl J Med.

